# Grounds for surrogate decision-making in Japanese clinical practice: a qualitative survey

**DOI:** 10.1186/s12910-020-00573-4

**Published:** 2021-01-13

**Authors:** Masashi Tanaka, Kayoko Ohnishi, Aya Enzo, Taketoshi Okita, Atsushi Asai

**Affiliations:** 1 Department of Medical Ethics, Tohoku University Graduate School of Medicine, Sendai, Japan; 2 Division of Clinical Epidemiology, National Hospital Organization Tokyo Medical Center, Tokyo, Japan; 3 Department of Nursing, Konan Women’s University, Kobe, Japan

## Abstract

**Background:**

In the coming years, surrogate decision-making is expected to become highly prevalent in Japanese clinical practice. Further, there has been a recent increase in activities promoting advance care planning, which potentially affects the manner in which judgements are made by surrogate decision-makers. This study aims to clarify the grounds on which surrogate decision-makers in Japan base their judgements.

**Methods:**

In this qualitative study, semi-structured interviews were conducted to examine the judgement grounds in surrogate decision-making for critical life-sustaining treatment choices in acute care hospitals.

**Results:**

A total of 228 participants satisfied the inclusion criteria, and 15 were selected for interviews. We qualitatively analysed the content of 14 interview transcripts, excluding one that did not meet the inclusion criteria. Based on this analysis, we extracted 4 core categories, 17 categories, 35 subcategories, and 55 codes regarding judgement grounds in surrogate decision-making. The four core categories were as follows: patient preference-oriented factor (Type 1), patient interest-oriented factor (Type 2), family preference-oriented factor (Type 3), and balanced patient/family preference-oriented factor (Type 4). The Type 4 core category represented attempts to balance the preferences of the patient with those of the surrogate decision-maker.

**Conclusions:**

Surrogate decision-makers based their decisions on important aspects related to a patient’s life, and they considered not only the patient’s preferences and best interests but also their own preferences. As the need for surrogate decisions will increase in the future, decision-makers will need to consider judgement grounds from a more diverse perspective.

## Background

In coming years, surrogate decision-making is expected to become highly prevalent in Japanese clinical practice. In 2017, Japan reported 1.34 million deaths, of which 70% corresponded with people of age ≥ 75 years. This number is predicted to continue increasing [1]. According to one report, 42.5% of hospitalised elderly people are required to make decisions about end-of-life treatment. However, only 29.7% have the capacity to make such decisions [2]. Therefore, the number of patients who rely on others to make decisions on their behalf (or surrogate decision-makers) is expected to increase.

In the United States, surrogate decision-makers are, in principle, expected to base their decisions on the substituted judgement standard, that is, they should consider what the patient, if he or she were competent, would choose [3]. Buchanan and Brock introduced “a hierarchy of standards” for surrogate decision-making, which include the following three standards: a patient’s known wishes, substituted judgements, and the patient’s best interests [4]. These standards provide guidance to surrogate decision-makers in making judgements and have to date been considered the ‘orthodox’ view of surrogate decision-making in bioethics [5].

However, as pointed out by many researchers [6], the grounds for making judgements in surrogate decision-making are not always based on such hierarchical standards. For instance, according to a Japanese study, some surrogate decision-makers and physicians base their decisions on their own preferences [7], which indicates that the patient’s preferences or best interests may not always form the grounds for judgements in surrogate decision-making. However, studies on this topic in Japan are limited in terms of sample size and content related to surrogate decision-making.

In recent years, activities to promote advanced care planning (ACP) have gained momentum in Japan. ACP is defined as ‘a process that supports adults at any age or stage of health in understanding and sharing their personal values, life goals, and preferences regarding future medical care’. For many, this process may include entrusting medical decision-making to another person or persons in case they cannot make such decisions on their own [8]. This may affect the manner in which judgements are made in surrogate decision-making. In 2018, the Ministry of Health, Labour and Welfare (MHLW) revised the ‘Guidelines on the decision-making process for end of life care’ [9] to reflect the increase in ACP-related activities and research efforts in Japan. The revised guidelines highlight the importance of discussing on a regular and repeated basis the patient’s intentions regarding medical and care strategies, as well as their desired way of living, under the premise that such intentions may change with variations in their physical and psychological conditions. In other words, the importance of ACP-related actions is emphasised in the revised guidelines. Another important point mentioned in the guidelines is that the patients themselves should specify a surrogate decision-maker who would presume their intentions before they become incapable of communicating their wishes [8, 9]. Further, the MHLW organises workshops for consultants across Japan as part of its jurisdictional project based on ‘Education for Implementing End-of-Life Discussion (E-FIELD)’ targeting medical practitioners. The goal of these workshops is to develop a consultation system involving approximately 400 medical institutions nationwide to promote decision-making that respects patients’ preferences [10]. The guidelines and workshops primarily advocate the use of the standards proposed by Buchanan and Brock in eliciting grounds for judgements and recommend the use of ACP. The dissemination of these guidelines can potentially change the judgement grounds in surrogate decision-making in Japan.

Cultural differences may affect the grounds for making judgements in surrogate decision-making. For example, whereas ACP is considered a process that respects patient preferences and has been actively implemented in Europe and the United States, some of its aspects such as the disclosure by physicians of the possible future deterioration of their patients’ condition to the patients themselves is culturally taboo in Japan. Moreover, with respect to the selection of a patient’s surrogate decision-maker, the Japanese culture values seniority (e.g. being the eldest male member of the family) over normative standards (e.g. being the one who knows the patient’s preferences better than anyone else). Differences in religious beliefs underlie such cultural differences, as well. The cultural and religious beliefs in Japan strongly endorse the sacredness of life and death, which promotes the notion that unnecessary suffering should be avoided [11]. Therefore, a consideration of such cultural perspectives in surrogate decision-making reveals that several aspects clearly differ between the surrogate decision-making practices followed in Japan and Western countries. However, culture is not a fixed concept, and the continuous promotion of ACP in Japan has been facilitating cultural transitions and the merging of various practices. Moreover, such transitions are not unique to Japan but are likely observed in other countries, as well. Therefore, this study on the current status of surrogate decision-making and ACP implementation in Japan has international relevance.

The purpose of this study is to clarify the grounds based on which judgements are made by surrogate decision-makers in Japan; such a clarification is particularly important since the country has a rapidly aging population, which has intensified activities related to surrogate decision-making. In particular, this study aims to reveal the judgement grounds in surrogate decision-making involving critical, life-sustaining treatment choices in acute hospitals. Finally, it clarifies how the spread of ACP practices may alter the judgement grounds in surrogate decision-making. To date, reports on surrogate decision-making in Japan have been limited to those published domestically. Hence, we consider it meaningful to report on the judgement grounds in surrogate decision-making that are currently being followed in Japan to an international audience.

## Methods

### Study design

Since each case was unique, we considered the need for research methods that enable the careful analysis and examination of each decision-making process to understand the reality of each case. Accordingly, we adopted a qualitative research design based on semi-structured interviews.

### Eligibility criteria

In this study, participants were recruited through a recruiting company from among individuals residing in the suburbs of Tokyo [12]. Eligible participants were the surrogate decision-makers of patients who satisfied all the following conditions: they (a) were hospitalised during the period spanning 1 April 2012 through 31 March 2017, (b) were incapable of making decisions for themselves, (c) were ≥ 65 years of age, (d) required someone to make decisions regarding life-sustaining treatment (dialysis, artificial respirator usage, tube feeding, or central venous hyperalimentation), and (e) held discussions regarding treatment with physicians in the presence of another individual. Further, the exclusion criteria were as follows: (a) they did not wish to participate in the study, (b) they had difficulty communicating in Japanese, and (c) they were underage. Based on the study protocol and an informed consent form, the recruitment company created a list of participants from a database in accordance with the inclusion and exclusion criteria. From this list of 228 people, the interviewer selected 15 participants and contacted them through the recruiting company. The protocol did not specify a limit to the number of individuals to be interviewed and, thereby, allowed for the possibility of conducting additional interviews. However, no additional interviews were necessary, since content saturation and theme development were achieved with the selected 15 participants. Further, the recruitment company provided to the investigators a list of the contact information of those who wanted to participate in the study.

### Interview procedure

The contents of planned interviews were explained to participants in the written form in advance. Researchers conducted interviews at the date and time specified by the interviewees in a conference room of the researchers’ study centre (Tokyo Medical Centre) and ensured participant privacy. The interviews were conducted in line with the questions listed in the interview guide (Table 1).

**Table 1:** Guide for interviews with surrogate decision-makers

1	What was the specific content(s) of your surrogate decision-making?
2	In surrogate decision-making, were you aware of the fact that you were making decisions as the patient’s “surrogate”?
3	Do you think making surrogate decisions was difficult for you?
4	Do you think your surrogate decision-making went smoothly?
5	Do you think there was a disagreement between the surrogate decision-maker and medical personnel or among several surrogate decision-makers?
6	Did you receive sufficient information from medical personnel when you made surrogate decisions? Do you think you were able to have meaningful discussions with medical personnel?
7	What were the bases for judgment in your surrogate decision-making?
8	After having performed surrogate decision-making, do you think you made a good surrogate decision for the patient?
9	Do you have any regrets regarding surrogate decision-making?

### Ethical considerations

This study was approved by the Ethics Committee of Tohoku University School of Medicine (Approval No. 2017-1-856). At the time of conducting the survey, the method and intent of the survey were explained to participants. Further, all the participants provided written informed consent and were assured that the contents of the interviews would be recorded and that their statements would be reported anonymously. All the participants were compensated 8000 yen (for approximately 30 min of paperwork and transportation expenses) for the interviews, which lasted up to 2 h.

### Analysis methods

Interview transcript preparation was outsourced to Kyoto Data Service, a private transcription company. One of the authors (MT) performed all the fidelity checks for voice audio data and transcript reports. Further, data were analysed using a qualitative analysis method that was based on the KJ method [13] and the Ueno method [14], which is a simplified version of the KJ method developed by Chizuko Ueno (Professor Emeritus, University of Tokyo).

Interviews were recorded using an IC recorder, and verbatim transcripts were created. All the researchers carefully read and analyzed each sentence in the raw data. The sentences or portions of sentences having the same content were coded. At the time of coding, no attempt was made to simplify expressions. Further, codes with similar content were grouped into a subcategory and given a name that represented the shared content. While creating subcategories, efforts were made to simplify their names so that the subcategory’s meaning could be readily understood from its name alone. Further, similar subcategories were grouped into categories and then into core categories, with increasing levels of abstraction. Authors verified the data’s content validity and analyses’ reliability and validity by conducting discussions until they reached a consensus regarding classification, as well as coding. The analyses were performed by a multidisciplinary group of researchers, including two physicians, one nurse, two philosophers, and one pharmacist.

Since the codes (sentences) derived using this analysis method were long, all of them could not be presented in this paper. Accordingly, the important parts of each code were excerpted, as appropriate, and some parts omitted without changing the overall meaning. Data were analysed using MAXQDA Plus12 (Release 12.2.1) software.

## Results

### Overview of study participants

A total of 228 respondents satisfied the inclusion criteria. Among them, 15 participants were selected from among the respondents who were available for interviews at a date and time convenient for both researchers and participants (i.e. interviewees). The number of individuals pooled by the web survey company [12], to whom survey requests could be sent, was not disclosed by the company. The 15 participants were interviewed over the course of six non-consecutive days such that up to three participants were interviewed per day in November and December 2017. Although 15 participants were interviewed, the data from only 14 were subjected to analysis; the remaining one participant’s data were excluded since the participant was a patient’s family member but did not make decisions on the patient’s behalf. Table 2 summarises the participants’ characteristics. All the surrogate decision-makers who participated in this study were family members of the respective patients.

**Table 2:** Characteristics of surrogate decision making in this research

Sex of surrogate decision-maker	Age of surrogate decision-maker	Relationship with patient	Interview duration (min)	Sex of patient	Age of patient	Cohabiting (patient and surrogate decision-maker)	Inpatient department	Content of surrogate decision-making				
								Life-prolonging treatment	Ventilator	Artificial nutrition	Place of treatment	Dialysis
Female	30s	Daughter-in-law	48	Male	70s	No	Cardiology	○	○	○	○	○
Female	40s	Eldest daughter	50	Male	70s	No	Neurology			○		
Female	40s	Daughter-in-law	40	Male	70s	No	Respiratory	○	○			
Male	60s	Second son	50	Male	80s	No	Neurology	○		○	○	
Male	60s	Eldest son	26	Female	90s	No	Internal medicine	○	○			
Female	40s	Eldest daughter	40	Male	80s	No	NEUROLOGY			○	○	
Male	40s	Eldest son	25	Male	60s	No	Cardiology		○	○		
Female	50s	Daughter-in-law	45	Female	70s	No	Internal medicine	○			○	
Female	50s	Second daughter	37	Male	80s	No	Internal medicine			○		
Female	40s	Second daughter	34	Male	70s	No	Neurosurgery			○	○	
Female	50s	Daughter in law	48	Female	70s	YES	Neurosurgery	○		○	○	
Female	60s	Second daughter	37	Female	70s	Yes	Internal medicine	○	○	○	○	
Male	60s	Eldest son	37	Female	80s	Yes	Internal medicine			○		
Male	50s	Eldest son	54	Female	80s	No	Internal medicine	○	○	○		○

### Qualitative analysis

Table 3 summarises the results of the qualitative analysis of the judgement grounds for surrogate decision-making in Japan. It clarifies that the study extracted 4 core categories, 17 categories, 35 subcategories, and 55 codes (in the following text, core categories, categories, subcategories, and codes are represented using quotation marks, square brackets, angle brackets, and parentheses, respectively).

**Table 3:** Results of the qualitative analysis of the judgment grounds for surrogate decision-making in Japan

Core category	Category	Subcategory
1. Patient preference-oriented factor	I respected the patient's preferences	I told them that I had been instructed by the patient to decide against life support
		Since the patient’s preferences were clear, my decisions never wavered
		I made the decision respecting the patient’s intention regarding life-prolonging treatment
	I respected the patient's presumed intentions	I made the decision based on my understanding of what the patient would do
		My daily communication helped (patient-family)
		I thought the family would be able to guess the patient’s intentions
		I think I made the decision believing it to be in line with the patient’s intentions
		I guessed the patient’s intentions by observing his/her condition
2. Patient interest-oriented factor	I tried making the decision by considering the patient’s best interests	I valued the patient's safety
		I thought it would be good for the patient to receive medical treatment and recover
		I did not know what was good for the patient
	I did not want to do anything cruel to the patient	I decided against life-prolonging treatment out of pity
		The patient appeared to be suffering; so, I thought he or she would be better off with gastrostomy
	I made the decision based on the patient’s ADL and communication capacity	I agreed to forego life-prolonging treatment because I sympathized with the patient when I saw him/her being bedridden
		I did not choose gastrostomy since the patient was unable to communicate
		I thought the patient would find it painful to live in a vegetative state
		I thought it was my ego that wanted to choose life-prolonging treatment when the patient's condition was such that no communication was possible
	I expected the patient to recover	If the patient had a chance at recovery, I wanted him/her to be treated
		I continued to hope for the patient's recovery
		I chose artificial alimentation in hopes of recovery
3. Family preference-oriented factor	I wanted to protect my family’s life and interests	I wanted to bring him/her home; so, I chose the procedure (gastrectomy)
		I judged it realistically impossible to provide home care
		I made the decision that family members would not regret
		I realistically considered the lives of family members and decided to forego gastrostomy
		I thought that the patient's safety would ensure my own self-protection since I was the surrogate decision-maker
	I made the decision based on the thoughts of family members and other people close to the patient	I ignored the discussions that we had in advance
		The feelings of the patient's closest family members were important
	I wanted the patient to live	I was aware of the stance of the patient who refused life support; but I wanted him/her to live
		When death suddenly became a real possibility, I, as a family member, wanted to prolong the patient’s life
	I wanted to do everything I could	I had the patient undergo gastrostomy for my family
		I thought nobody would want to die
		Because the patient's life was limited, I wanted to keep him/her alive for one more day
	I accepted death	I had no regrets; so, I didn’t choose life support
		I thought that death was inevitable
4. Balanced patient/family preference-oriented factor	I balanced the patient's intentions and lives of family members	I made the decision considering the balance between the patient's life and the lives of family members
		I balanced the intention of the patient and the thoughts of family members

#### Type 1: Core category ‘Patient preference-oriented factor’

The Type 1 core category included the judgement grounds rooted in the patient’s preferences. This core category comprised 2 categories, 8 subcategories, and 13 codes. Some representative categories, subcategories, and codes are as follows:

[I respected the patient’s preferences].

One of the subcategories under this category was < Since the patient’s preferences were clear, my decisions never wavered >, which included the following code: (I had conversations with the patient in advance. We often half-jokingly talked about when the patient was going to die. The patient also mentioned specific matters, such as not wanting to live with various machines connected to the body). In this case, the patient mentioned specific treatment choices in prior discussions, and the surrogate decision-maker respected these choices while making decisions.

[I respected the patient’s presumed intentions].

One of the subcategories under this category was < I made the decision based on my understanding of what the patient would do >, which included the following code: (We, as family members, tried to put ourselves in the patient’s place. We wondered which one of the choices my father would make after hearing what the doctor had said, had he been able to make his own decision). In this case, the surrogate decision-maker attempted to determine the patient’s preferences from the patient’s perspective.

#### Type 2: Core category ‘Patient interest-oriented factor’

This category indicated the judgement grounds rooted in the patient’s interests. This core category comprised 4 categories, 12 subcategories, and 20 codes. Some representative categories, subcategories, and codes of this core category are as follows:

[I tried making the decision by considering the patient’s best interests.

This category included the subcategory < I thought it would be good for the patient to receive medical treatment and have an opportunity to recover >, which contained the following code: (What I thought would be good for the patient was, for example, to be able to lead a normal life as before, even if it is somewhat inconvenient. I thought any decision that would facilitate this would be in the patient’s best interest and a good decision). The case in which a surrogate decision-maker considers a treatment option that enables the patient to live as usual is in line with the patient’s best interests and uses the patient’s best interests as the basis for decision-making.

[I did not want to be cruel to the patient].

This category included the subcategory < I decided against accepting life-prolonging treatment out of pity for the patient >, which contained the following code: (To be honest, we as family members just felt sorry for the patient, whom we couldn’t even recognize anymore, and since we were no longer able to have a conversation, we did not know how much the patient could understand what we were saying—so, we did not choose life-prolonging treatment. We clearly communicated these thoughts to the doctor and made the decision). The surrogate decision-maker judged that the treatment could not preserve the patient’s dignity once the latter’s condition worsened. This formed the basis of the decision-maker’s decision to tell the physician that life support was not desired.

[I made the decision based on the patient’s activities of daily living (ADL) and communication capacity].

This category included the subcategory < I thought the patient would find it painful to live in a vegetative state >, which contained the following code: (I might come off as an ungrateful child if I say this, but my feeling was that, rather than living in a vegetative state at age 87, the patient would be better off just dying. … Living in pain, being connected to numerous tubes, just lying in bed, and sleeping for 1 year or 2 years—how pitiful, I thought, if that’s what it comes to). The surrogate decision-maker felt sorry for the patient living with significantly low ADL at an advanced age. Such thoughts can cause the decision-maker to make a decision that shortens the patient’s time to death. Further, this code reflected a sense of guilt associated with making a surrogate decision based on the family’s preferences.

#### Type 3: Core category ‘Family preference-oriented factor’

Type 3 core category included the judgement grounds rooted in the preferences of the surrogate decision-maker, who is a member of the patient’s family. This core category comprised 5 categories, 13 subcategories, and 17 codes. In this category, surrogate decision-makers made decisions on behalf of the patient based on their own (their family’s) preferences, rather than considering the patient’s preferences. Whereas the surrogate decision-maker was unaware of the patient’s preferences in some cases, he or she was aware of the patient’s preferences but chose not to consider them and prioritised their own preferences in other cases.

[I wanted to protect my family’s life and interests].

This category included the subcategory < I realistically considered the lives of family members and decided to forego gastrostomy >, which contained the following code: (I thought, ‘I must look to the best interests of my father’, but realistically speaking, my younger sister, the second daughter, had young children and was running her own business. Her life would have been affected if she did not work. As the eldest daughter, I myself was also unable to leave the house for a long period of time because I was raising my children. Therefore, it was not at all realistic for us to provide home care. I shut my eyes to his pain and wishes and decided that he should not receive gastrostomy in consideration of continuing his medical treatment at the hospital). Although this surrogate decision-maker wished to prioritise the patient’s preferences, she made a decision that did not adhere to the patient’s preferences in consideration of the realistic circumstances surrounding herself, as well as other family members.

[I made the decision based on the thoughts of family members and people close to the patient].

This category included the subcategory < The feelings of the patient’s closest family members were important >, which contained the following code: (I needed to convince my mother-in-law, who was closest to the patient. I thought that, rather than us (the son and his wife) making decisions against her will, she should make decisions that are satisfactory to her once she has organised her own thoughts. For this reason, it took a lot more time to come to a decision, and I’m afraid my father-in-law suffered for a long period). This code describes a surrogate decision-making process in which the surrogate decision-maker secured the time necessary for the family to agree with the decision. However, this, in turn, increased the time that the patient was in pain.

[I wanted the patient to live].

This category included the subcategory < When death suddenly became a real possibility, I, as a family member, wanted to prolong the patient’s life >, which contained the following code: (The shock was tremendous when the doctor told us that death was inevitable, as the patient’s condition worsened. At that time, I honestly just thought, ‘I want the patient to live, even a day longer’, and it didn’t matter if gastrostomy, or anything, had to be done. It was hard for the family to say goodbye all of a sudden; so, I wanted the patient to get better, even just a little. I was always prepared, to no small extent. But when a doctor talks about life or death, you can’t help but think ‘please just help the patient’). When the patient’s death became a real possibility due to the worsening of his or her health condition, the desperate hope of the surrogate decision-maker to prolong the patient’s life formed the basis of judgement in surrogate decision-making.

#### Type 4: Core category ‘Balanced patient/family preference-oriented factor’

The Type 4 core category included judgement grounds rooted in balancing the preferences of both the patient and the surrogate decision-maker (i.e. family). This core category comprised one category, two subcategories, and five codes.

[I balanced the patient’s intentions and lives of family members].

This category included the subcategory < I made the decision considering the balance between the patient’s life and the lives of family members >, which contained the following code: (I had mixed feelings when I had to make a decision on the patient’s nutrition. Considering the burden on my brother and his wife who were actually providing care, I wondered how my decision might affect their lives. On the other hand, I also had to think about the feelings of my father who wanted to recuperate at home. It was a hard decision to make. I was particularly worried about the burden on my sister-in-law). As suggested by this code, the surrogate decision-maker made decisions by considering the patient’s wish to receive home care and the burden on the lives of the family members who provided the care. Based on these considerations, the surrogate decision-maker ultimately decided on gastrostomy as a means of nutrition support, which was not in line with the patient’s wish to receive home care. This decision also aimed to reduce the burden of care on family members.

## Discussion

This study identified four types of judgement grounds for surrogate decision-making in Japan. In view of the standards proposed by Buchanan and Brock to guide surrogate choices, the Type 3 (family preference-oriented) factor must be avoided to the maximum extent possible in the reasoning process leading to surrogate decisions. Further, our findings clarify the difficulty involved in eliminating this factor. In the following sections, we include an analysis of this factor from a cultural perspective and discuss the influence of ACP, which is expected to become widespread in Japan in the future.

### Culture in which conversations about end of life rarely occur

One of the subcategories extracted in this study was < I did not know what was good for the patient > . This subcategory reflects the struggle experienced by the surrogate decision-maker in making decisions since no matter how hard the surrogate decision-maker tries to guess the patient’s preferences, there is no way of knowing the patient’s actual choices. During the interviews, surrogate decision-makers described difficulties in presuming the patient’s preferences, which suggests that it is difficult for surrogate decision-makers to see things from the patient’s perspective.

One factor that contributes to this difficulty is the cultural taboo on specific conversations about the end of life (EOL). Such talk is generally considered bad luck and even taboo for some families. Only 5.5% of Japanese citizens reportedly talked about medical treatment in EOL situations with their family or medical care personnel, and only approximately 8% put their intentions in writing beforehand [15]. Hence, the number of surrogate decision-makers who clearly recognise their patients’ preferences is likely to be low.

### Changes in social circumstances

In 1980, approximately 70% of the elderly people aged ≥ 65 years lived with their children. By 2015, this rate had significantly declined to 39.0%. However, the rate of double-income families, which was 49.3% in 1980, has been increasing annually, reaching 64.4% in 2015 [16]. These data suggest an increase in the number of adults (i.e. the offspring of elderly individuals) who cannot stay home all day. In terms of the communication between parents and children who do not live in the same house, Japan reportedly has a lower frequency of older individuals meeting or calling their non-cohabiting children compared to the United States, Germany, and Sweden [17]. Although these comparisons are made among a small number of countries, international averages confirm the low frequency of communication between elderly individuals and their non-cohabiting children or other family members in Japan.

In recent decades, it has become less common for children (i.e. potential surrogate decision-makers) to share time and space with their parents (i.e. patients) on a daily basis. Hence, the children in the current generation likely experience difficulty in understanding or imagining how their parents live or what they value in their daily living. This may present an obstacle when they attempt to make a surrogate decision and may also underlie the basis of their judgements in surrogate decision-making that involve factors other than the patient’s preferences or best interests. All the 14 surrogate decision-makers considered in this study were children of patients or the spouses of such children. Although information regarding their work status was unavailable, the rate of cohabitation with patients was low (20%), which may have made it difficult for these surrogate decision-makers to envisage and understand their patients’ lives and values.

### Time restrictions in surrogate decision-making

Time restrictions likely influenced the judgement grounds in surrogate decision-making. According to a report from the United States, 48% of surrogate decision-makers had to make critical decisions about life-sustaining treatment for patients aged ≥ 65 years within 48 h after hospitalisation in acute hospitals [18]. This implies that surrogate decision-makers may be forced to make decisions quickly, particularly in acute care settings. In such a scenario, to what extent would the surrogate decision-maker consider the patient’s preferences in their judgements? Some families in the current study chose to forego life-prolonging treatment for the patient (e.g. < I judged it realistically impossible to provide home care >). Settings that require judgements on treatment options related to life support will likely affect the life of the surrogate decision-maker (family) to some extent depending on the treatment’s outcome, particularly when the patient’s condition is unfavourable. In such situations, the decision-maker may make a hasty decision about treatment choices that reflects the inability of the surrogate decision-maker to bear the burden of care because of their own life circumstances.

### Novelty of the Type 4 factor

The Type 4 factor reflects the reasoning of surrogate decision-makers who make an effort to balance the preferences of the patient and those of family members. Earlier studies on judgement grounds in surrogate decision-making only introduced only a single basis of judgement for each case of surrogate decision-making, that is, they discussed only one factor that affected decision-making. In contrast, the current study identified three factors that are not necessarily mutually exclusive; this suggests the possibility that multiple elements might be involved in the reasoning and derivation of surrogate decisions in actual decision-making. We analysed interview contents that spanned the entire process of surrogate decision-making up to the judgement stage, which yielded numerous judgement grounds for each case of surrogate decision-making. Further, we extracted 55 codes from our analysis of the 14 cases. We speculate that these codes are considered in combination in surrogate decision-making settings, perhaps even in a comparative manner. Codes related to the Type 4 factor were categorised separately from those related to Types 1–3, since Types 1–3 reflect a single judgement ground, whereas Type 4 reflects the outcome of the comparative weighing of multiple grounds. In the United States, where patient autonomy is valued, surrogate decision-makers have been reported to derive decisions based on their own values and circumstances in some cases [19, 20]. However, the current study is the first to address this issue in Japan.

### Concerns about potential psychological difficulties in surrogate decision-making with the spread of ACP in Japan

ACP is suggested to strengthen the communication between physicians and surrogate decision-makers [21]. Although the widespread implementation of ACP is considered beneficial to patients, there are concerns that the widespread use of ACP may complicate the process of surrogate decision-making and increase the psychological burden on surrogate decision-makers. ACP may necessitate surrogate decision-makers to place more weight on patient preferences while making decisions, which may increase the burden on decision-makers because there are situations where surrogate decision-making should be based on the preferences of the surrogate decision-maker, which may differ from those of the patient.

In Japan, patients rarely talk about their own treatment preferences and values. Having advance discussions with the patient more often will allow the surrogate decision-maker to be more aware of the patient’s preferences than they have been in the past and may help them identify judgement grounds that are rooted in the patient’s preferences and best interests. However, this may also lead to a clear awareness that the preferences of decision-makers differ from those of the patient. In this manner, surrogate decision-makers may become more conflicted in their struggle to decide whether to prioritise their own or the patient’s preferences. Further, the clarification of patient preferences through ACP might not necessarily result in the prioritisation of these preferences, but instead place a psychological burden on surrogate decision-makers who must struggle to balance the preferences of both the patients and their own families. A discussion on whether such a struggle is beneficial or not to all concerned is beyond the scope of this discussion. Nonetheless, it could complicate the process of surrogate decision-making. Rather than focusing solely on the principle of respect for patient autonomy and decision-making standards, judgement grounds in surrogate decision-making should be discussed while considering multiple factors, including the cultural context, social context, and circumstances of the surrogate decision-makers. Further, earlier studies have reported that some patients permit the balancing of their preferences with those of surrogate decision-makers (6.22).

In some cases, patients may prefer to be aware of potential conflicts before choosing their surrogate decision-makers. ACP discussions can help a patient choose a different surrogate decision-maker who may not have conflicts or who may be more willing to abide by the patient’s wishes. We believe that our results are not only helpful to healthcare professionals in Japan but also widely applicable to countries with similar cultural values, for instance, countries that do not prioritise patient self-determination to the extent done by the United States and countries that consider the interests of the individuals surrounding the patient, such as his or her family members. Even in Western countries, not all patients perform ACP, and not all want their autonomy to be respected above all else [23]. If a healthcare professional encounters cases similar to those discussed in this paper, the findings of this study can provide meaningful information that contributes to surrogate decision-making.

### Strengths and limitations of the study

The analyses in this study were performed by a multidisciplinary group of six professionals, which included non-medical practitioners (two physicians, one nurse, two philosophers, and one pharmacist), as well. This enabled discussions from various perspectives, which contributed to a well-rounded analysis in contrast with the narrower strategies adopted in earlier studies. While developing the analysis method, we considered the Ueno method, which is based on the KJ method. This enabled us to analyse the entire process of surrogate decision-making and comprehensively identify the judgement grounds. Further, the Ueno method is superior to other methods in that it facilitates the analysis of the entire interview content without omitting any information.

This study has some limitations, as well. First, since the recruitment process was outsourced to a web research company, interview respondents were limited to Internet users living in the suburbs of Tokyo near the location of the interview site. Potential bias also exists because detailed information on the characteristics of surrogate decision-makers, such as their number of years of care experience, educational background, economic status, and religion, and the family composition of patients, other than the surrogate decision-maker, was not available. However, since the data from 14 participants were sufficient to achieve the theoretical saturation of concepts extracted as judgement grounds in surrogate decision-making, we did not increase the sample size.

Second, recall bias may have occurred due to the timing of the interviews. The interview survey was performed within 6 months to 3 years after the surrogate decisions were made. Due to this time lag, the interview contents might have differed from actual events. However, since experiences of surrogate decision-making are often connected to grief, consideration was given such that the interviews were conducted after a certain amount of time had passed.

Finally, although the Ueno method has some analytical advantages, it has not been validated internationally. Hence, no English description for this method is available, and no studies using this method have been reported internationally.

Despite these limitations, the current study provides novel insights into the grounds for making judgements in surrogate decision-making. We further suggest that a large-scale cross-sectional study on this topic can further clarify the diversity and frequency of judgement grounds in surrogate decision-making in Japan.

## Conclusions

This study revealed the current state of surrogate decision-making in Japan. When making decisions on important aspects related to a patient’s life, surrogate decision-makers based their decisions on not only the preferences and best interests of the patient but also their own and their family’s preferences. The bases underlying the preferences of surrogate decision-makers included their own perspectives of life and death, values, and care burden.

ACP will likely become more prevalent in Japan in the future. It is a valuable source of information and helps respect patient autonomy. However, due to the cultural and social backgrounds of Japan, it is unclear whether this practice can be appropriately reflected by the judgement grounds in surrogate decision-making. Further, as discussed earlier, basing judgements solely on the principle of respect for patient autonomy or the standards of surrogate decision-making that originated in the United States would be undesirable in the Japanese context. Therefore, in Japan, the making of surrogate decisions based on judgement grounds from diverse perspectives appears to be more appropriate.

## Data Availability

The datasets used by and/or analysed during the study are available from the corresponding author upon reasonable request.
